# Cardiac Rhabdomyomas Presenting with Critical Cardiac Obstruction in Neonates and Infants: Treatment Strategies and Outcome, A Single-Center Experience

**DOI:** 10.1007/s00246-024-03420-0

**Published:** 2024-03-13

**Authors:** Li Yen Ng, Jonathan McGuinness, Terence Prendiville, Orla Franklin, Mark Walsh, Damien Kenny, Lars Nolke, Colin J. McMahon

**Affiliations:** 1 Department of Paediatric Cardiology, Children’s Health Ireland at Crumlin, Dublin, 12 Ireland; 2 Department Paediatric Cardiology, Department of Congenital Cardiothoracic Surgery, Children’s Health Ireland, Crumlin, Dublin, 12 Ireland; 3 UCD School of Medicine, Belfield, Dublin, 4 Ireland; 4 Maastricht School of Health Professions Education, Maastricht, The Netherlands

**Keywords:** Cardiac rhabdomyomas, Primary cardiac tumor, Tuberous sclerosis complex, Mammalian target of rapamycin inhibitor

## Abstract

Cardiac rhabdomyomas are the most common benign pediatric heart tumor in infancy, which are commonly associated with tuberous sclerosis complex (TSC). Most rhabdomyomas are asymptomatic and spontaneously regress over time. However, some cases especially in neonates or small infants can present with hemodynamic instability. Surgical resection of the tumor, which has been the gold standard in alleviating obstruction, is not always possible and may be associated with significant morbidity and mortality. Recently, mammalian target of rapamycin inhibitors (mTORi) have been shown to be safe and effective in the treatment of TSC. We present the outcomes of neonates and an infant who received treatment for symptomatic rhabdomyomas at a tertiary cardiology center. Medical records were reviewed to obtain clinical, demographic, and outcome data. Six patients received interventions for symptomatic rhabdomyomas, median age at presentation was 1 day old (range from 1 to 121 days old), and 67% of the patients had a pathogenic mutation in TSC gene. One patient underwent surgical resection of solitary tumor at right ventricular outflow tract (RVOT) successfully. In the four patients with left ventricular outflow tract (LVOT) obstruction, two patients received combined therapy of surgical debulking of LVOT tumor, Stage I palliation procedure, and mTORi and two patients received mTORi therapy. One patient with RVOT obstruction underwent ductal stenting and received synergistic mTORi. Four of the five patients had good response to mTORi demonstrated by the rapid regression of rhabdomyoma size. 83% of patients are still alive at their latest follow-up, at two to eight years of age. One patient died on day 17 post-LVOT tumor resection and Hybrid stage one due to failure of hemostasis, in the background of familial factor VII deficiency. Treatment of symptomatic rhabdomyoma requires individualized treatment strategy based on the underlying pathophysiology, with involvement of multidisciplinary teams. mTORi is effective and safe in inducing rapid regression of rhabdomyomas. A standardized mTORi prescription and monitoring guide will ensure medication safety in neonates and infants with symptomatic cardiac rhabdomyoma. Although the majority of tumors responded to mTORi, some prove to be resistant. Further studies are warranted, ideally involving multiple international centers with a larger number of patients.

## Introduction

Cardiac rhabdomyomas are the most common benign tumors of the heart in infancy [1]. While they may occur in isolation, these lesions are highly specific and pathognomonic for Tuberous sclerosis Complex (TSC). TSC is an autosomal dominant neurocutaneous genetic disorder caused by a mutation in TSC 1 or TSC 2 gene [1], which in turn causes over-activation of mammalian target of rapamycin (mTOR) pathway, leading to formation of multiple benign tumors or hamartomas within various organs, including the brain, kidney, and heart [2].

The natural history of the cardiac rhabdomyomas is well described, with an initial increase in tumor size and subsequent progressive reduction in size over time [3]. These lesions can arise from anywhere within the heart but most commonly in the ventricles. The tumors usually do not cause any serious medical problem. However, in some cases, especially in the neonates and young infants they may present in extremis with heart failure or hemodynamic instability due to high-tumor burden and/or mechanical obstruction of the inflow or outflow tract [4].

Echocardiography is the imaging modality of choice. On echocardiographic imaging, these lesions appeared to be echogenic, nodular masses that arise from within the myocardium. Cardiac magnetic resonance imaging can also be used to provide complimentary information, such as tissue characterization and evaluation of ventricular systolic function. Complementary imaging often plays a role in surgical planning where surgical resection is required [5].

Surgical excision of the tumor mass is indicated for hemodynamically significant lesions, including those lesions that obstruct the cardiac inflow or outflow tract. However, complete surgical excision of the tumor may not be always possible due to the risk of damage to nearby structures and a debulking procedure may be warranted [6].

Recently, mTOR inhibitors (mTORi) such as sirolimus and everolimus have been shown to be effective in the treatment of the TSC-associated tumors in the brain (subependymal giant cell astrocytoma [SEGA]), kidney (angiomyolipoma [AML] and lymphangioleiomyomatosis), and cardiac rhabdomyoma [7–10]. To date, there are single-case reports or small series reporting the effect of mTORi in cardiac rhabdomyoma [9, 11–15]. However, there is a lack of consensus for mTORi treatment dosing and strategy in cardiac rhabdomyoma [16].

We aim to present the management strategy and outcomes for neonates and infants presented with obstructive cardiac rhabdomyoma who required intervention at our center from 2014 to 2022. Second, we aim to identify the optimal dosing regimen and to formulate a guide for the prescription for mTORi, specifically sirolimus, in the treatment of symptomatic cardiac rhabdomyoma in neonates and infants.

## Methods

This retrospective study reports the treatment strategy and short-medium-term outcome of the neonates and infants presented with symptomatic cardiac rhabdomyoma at our center, from 2014 to 2022.

All neonates and infants with symptomatic cardiac rhabdomyoma (CR) who received mTORi therapy, surgical resection, or hybrid procedure [bilateral pulmonary artery (BPA) bands and ductus arteriosus (PDA) stenting] were included.

Medical records were reviewed for each patient for demographic and outcome data.

## Results

**Fig. 1:**
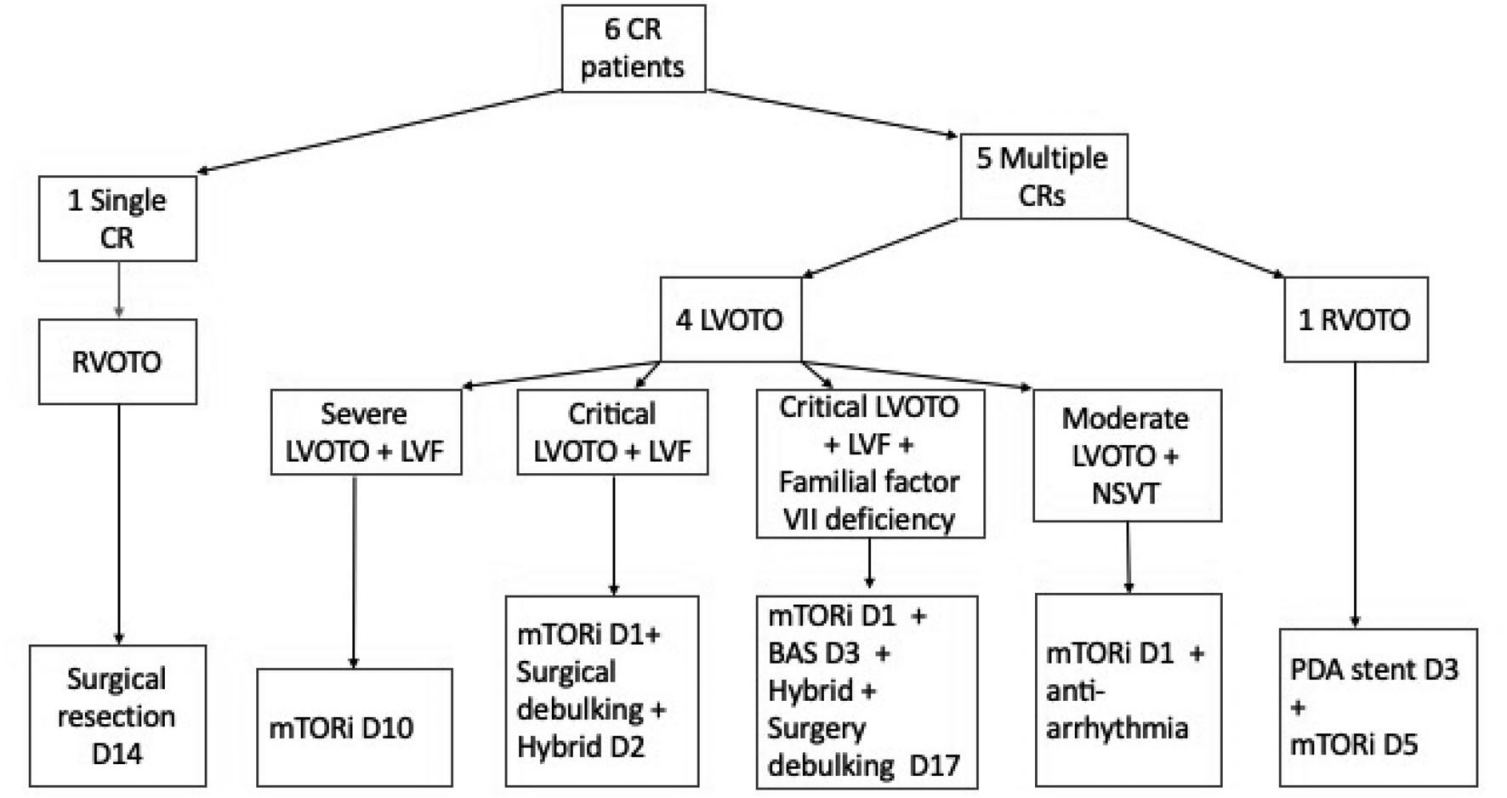
Cardiac Rhabdomyoma pathophysiology at presentation & treatment strategy. *CR* cardiac Rhabdomyoma, *RVOTO* right ventricular outflow tract obstruction, *LVOTO* left ventricular outflow tract obstruction, *LVF* left ventricular failure, *NSVT* non-sustain ventricular tachycardia, *D* day, *BAS* balloon atrial septostomy, *PDA* patent ductus arteriosus, *mTORi* mammalian target of rapamycin inhibitor

Table 1 and Flowchart 1 (Fig. 1) outline patient characteristics, clinical presentation, and treatment strategies for cardiac rhabdomyoma.

**Table 1:** Patient characteristics, presentation, and treatment strategy for cardiac rhabdomyomas

	Case 1	Case 2	Case 3	Case 4	Case 5	Case 6
Sex	M	F	M	F	M	M
Antenatal diagnosis (Y/N)	Y	N	Y	Y	N	Y
Gestation at birth, week + day	Term	Term	37 + 3	36 + 5	36 + 5	38 + 4
Age of diagnosis	At birth	6 days old	At birth	At birth	4 months old	At birth
Weight at diagnosis, kg	3	3.16	3.2	2.86	6	3.09
TSC gene mutation	TSC2 mutation	Not found	TSC2 mutation	TSC2 + PKD1 mutation	Not found	TSC mutation
Tumor burden	Multiple	Single	Multiple	Multiple	Multiple	Multiple
Location of largest of symptomatic CR	LVOT tumor	Single pedunculated CR at RVOT	Multiple CR in LV causing critical LVOTO & LVF	Subpulmonic CR tethering under surface of PV, protruding into RV	Interventricular septum into LVOT	Interventricular septum
Presentation/ Reason to intervene	Severe LVOTO (4.2m/s)	Cardiac murmur, severe RVOTO	Critical LVOTO (4m/s), LVF	Severe RVOTO (3.5m/s)	Aborted cardiac arrest, LVOTO (2.8m/s) & RV inflow partial obstruction; Ventricular arrhythmia	Critical LVOTO, LVF
Treatment Strategy	Sirolimus (mTORi)	Surgical resection of RVOT tumor DOL6	Surgical resection of LVOT tumor and Hybrid stage 1 on DOL2 + Sirolimus (mTORi)	Sirolimus (mTORi) + PDA stenting on DOL3	Sirolimus (mTORi) + antiarrhythmic	Sirolimus (mTORi) + Surgical resection of LVOT tumor and Hybrid stage 1 on DOL17

*M* male, *F* female, *Y* yes, *N* no, *TSC* tuberous sclerosis complex, *CR* cardiac rhabdomyoma, *kg* kilogram, *LV* left ventricle, *RV* right ventricle, *LVOT* left ventricular outflow, *RVOT* right ventricular outflow tract, *LVOTO* LVOT obstruction, *RVOTO* RVOT obstruction, *LVF* LV failure, *PV* pulmonary valve, *m/s* meter/second, *mTORi* mammalian target of rapamycin inhibitor, *DOL* day of life

Six patients [4 male] with CR received interventions; four cases of which were diagnosed antenatally. The median age at presentation was 1 day old (range from 1 to 121 days old), with symptoms varying from cyanosis and poor feeding to hemodynamic instability and aborted cardiac arrest. Two-thirds of the patients (4 of 6) were tested positive for a pathogenic mutation in TSC gene.

**Fig. 2:**
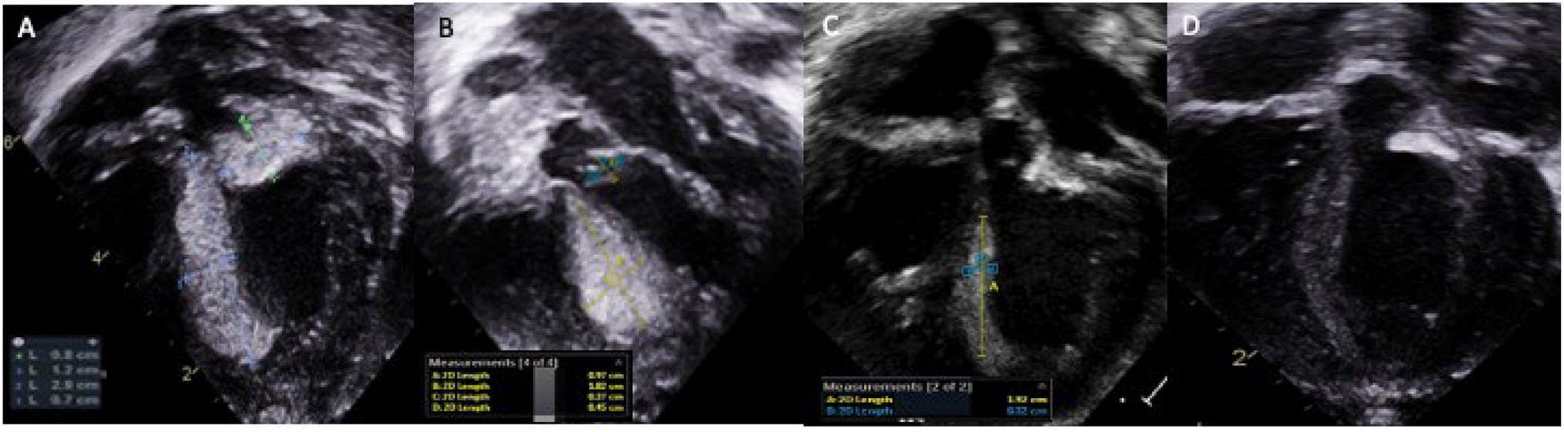
Serial images of Transthoracic Apical five-chamber view in case 2: **A** at birth; **B** 8 days post surgical resection of LVOT tumour and Sirolimus therapy; **C** 2 weeks post Sirolimus; **D** 4 weeks post Sirolimus. Note: at 2 weeks post mTORi, the tumour at interventricular septum (IVS) has regressed 35–55% compared initial presentation. Same tumour was completely regressed at weeks of Sirolimus therapy

**Fig. 3:**
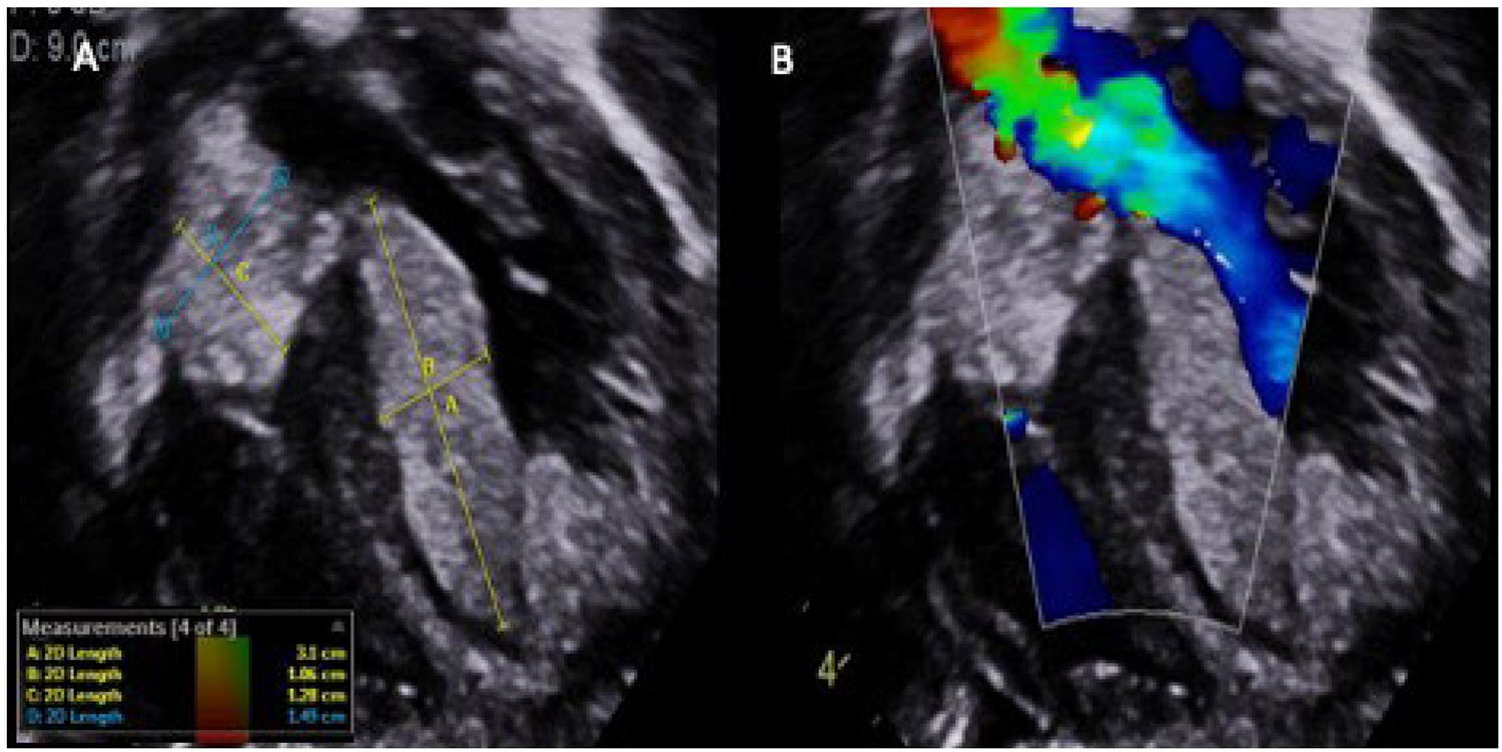
**A**&**B** Transthoracic Apical five-chamber (A5C) view with color Doppler compare on initial presentation in Case-5 showing multiple intracardiac rhabdomyomas in the left ventricle (LV) crowding the intraventricular cavity and left ventricular ouflow tract (LVOT) and a large tumour in the right atrium partially obstructing the right ventricular inflow

**Fig. 4:**
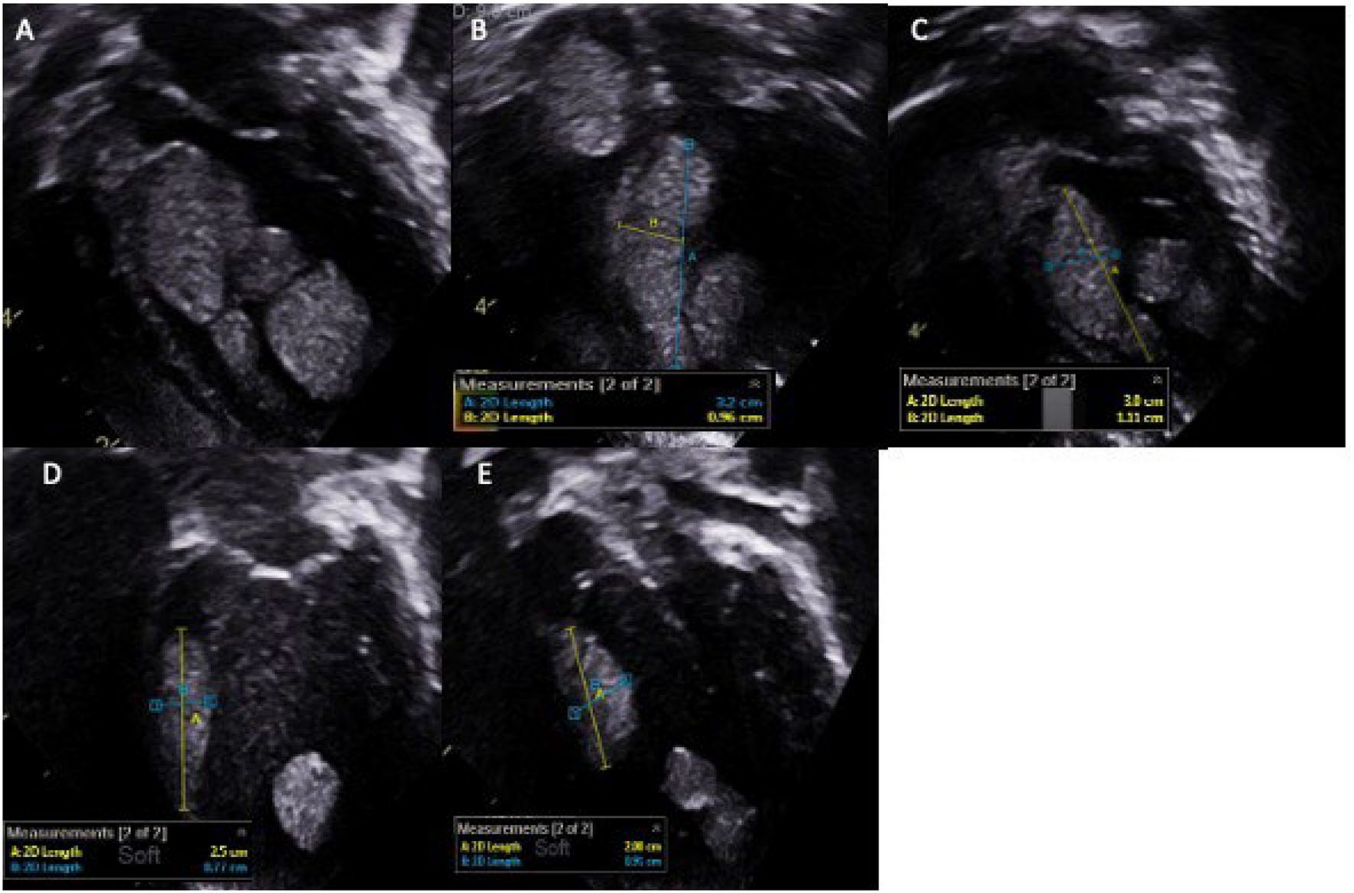
Serial Transthoracic Images in cas 5. **A** A5C view at 2-weeks, **B** A4C view at 4-weeks & **C** A4C view at 12-weeks of Sirolimus therapy showing minimal change in tumour size; **D**&**E** A4C & A5C views at 20-months after cessation of mTORi therapy showing 15–30% natural tumour regression

One of the six patients successfully underwent surgical resection of a solitary tumor in the right ventricular outflow tract. Five patients presented with multiple tumors. Four of the five patients had hemodynamically significant left ventricular outflow tract obstruction (LVOTO) (Figs. 2, 3, 4). Two of the four patients had critical LVOTO and severe left ventricular (LV) failure and were treated with surgical excision of LVOT tumor, palliative stage 1 procedure to use right ventricle to support the systemic circulation, and synergistic mTORi therapy. One of the four patients with severe LVOTO was treated with mTORi only and the other one of the four patients with moderate LVOTO and ventricular arrhythmia were treated with mTORi and antiarrhythmics. The last patient with multiple tumors presented with severe right ventricular tract obstruction (RVOTO) and was treated with mTORi and ductal stenting to maintain pulmonary circulation.

Table 2 outlines sirolimus dosing and monitoring strategy and Table 3 outlines clinical outcomes.

**Table 2:** Sirolimus (mTORi) strategy at our centre—dosing & monitoring

	TSC mutation	Sirolimus starting dose	Timing of 1st mTORi level, Day post 1st dose of mTORi	1st serum mTORi level, ng/mL (Target 15–20)	Side effects
Case 1 DOL1 ♂ Ex-term	TSC2	0.5mg OD (2.3mg/m^2^ OD)	7	26*	↑lipid
Case 3 DOL1 ♂ Ex-37 + 3	TSC2	0.5mg OD (2.3mg/m^2^ OD)	7	108**	↑lipid, AKI
Case 4 DOL1 ♀ Ex-36 + 5	TSC2 + PKD1 deletion	0.2mg OD (1mg/m^2^ OD)	4	47.8***	No
Case 5 4/12 ♂ Ex-36 + 5	No TSC mutation	0.3mg OD (0.9mg/m^2^ OD)	3	5.5^§^	No
Case 6 DOL1 ♂ Ex-term	TSC	0.3mg OD (1.4mg/m^2^ OD)	7	92^¶^	↑lipid

*DOL* day of life, ♂ male, ♀ female, *TSC* tuberous sclerosis complex, *PKD* polycystic kidney disease, *mg* milligram, *AKI* acute kidney injury, *OD* once daily, *mTORi* mammalian target of rapamycin inhibitor, ↑ increased, * mTORi dose reduced to 0.4mg OD, ** dose held and restarted once mTORi level with therapeutic level 2 weeks later at 0.4mg OD, *** mTORi dose stopped and restarted at 3 months old for rebound growth and worsen right ventricular outflow obstruction, § frequent up titration, ¶ mTORi dose held awaiting level to drop within therapeutic range

**Table 3:** Clinical Outcome of Neonates and Infant with Cardiac Rhabdomyomas

	TSC mutation	mTORi duration	Effect of mTORi on largest CR regression	Other TSC manifestation	Age & CR volume at the latest follow up
Case 1	TSC2	24 days	↓33–54% by day5; ↓70% by day24	SE nodules on MRI brain, Epilepsy	8 years old. No residual CR
Case 2	No TSC mutation	N/A	N/A	Nil	6 years old. No residual CR
Case 3	TSC2	4 years	↓10–40% by day8; ↓100% by 4 weeks	SE nodules on MRI brain, Epilepsy, learning needs	4 years old. Small residual CRs in RV & papillary muscles. Normal BVSF
Case 4*	TSC2 + PKD1 deletion	4 days + 3 months	↓25–30% by 2 weeks; Restarted at 3/12 old for rebound, ↓40–70% by 4 weeks	SE nodules in brain and cystic changes in kidneys	3 years old. Small residual RV CR. Normal BVSF
Case 5	No TSC mutation	3 months	Minimal response to mTORi despite frequent up titration	Nil	2 years old. Multiple CRs in LV but ↓15–30%, no cardiac obstruction. Normal BVSF
Case 6	TSC	7 days	↓20–50% by day8	Nil	Died on 17 days old due to post operative complication

*TSC* tuberous sclerosis complex, *PKD* polycystic kidney disease, *CR* cardiac rhabdomyoma, *mTORi* mammalian target of rapamycin inhibitor, *MRI* magnetic resonance imaging, ↓decreased, * mTORi dose stopped and restarted at 3 months old for rebound growth and worsen right ventricular outflow obstruction, *SE* subependymal, *LV* left ventricle, *RV* right ventricle, *BVSF* biventricular systolic function, *N/A* not applicable

Sirolimus (mTORi) prescription, monitoring strategy, and clinical outcome: We observed a variable starting dose for mTORi, from 0.9 to 2.3 mg/m^2^/day, with variable timing of serum drug level after 1st dose, from day 3 to day 7. Four of the five patients have documented supratherapeutic serum mTORi level, from 26 to 108 ng/mL (therapeutic target range = 15–20 ng/mL). Four of the five patients had good response to mTORi demonstrated by the rapid regression of rhabdomyoma size.

Eighty-three percent of (5 of 6) patients are still alive at their latest follow-up, between two and eight years of age. One patient died on day 17 post-LVOT tumor resection and Hybrid stage one due to failure of hemostasis, with a background of a familial factor VII deficiency.

Two of the four patients with TSC mutation have small residual rhabdomyomas and manifestations of TSC in brain and/or kidneys at their latest outpatient visits. Of the two patients without TSC mutation, one has no residual cardiac rhabdomyoma and the other patient has spontaneously regressing cardiac rhabdomyoma despite ongoing asymptomatic ventricular arrhythmia.

## Discussion

This case series demonstrates that good clinical outcomes are possible in the management of hemodynamically significant cardiac rhabdomyomas even in the hearts of small neonates and a young infant. This was achieved using a personalized patient-specific treatment strategy. The variable pathophysiology at presentation (see Table 1) were due to variable tumor location and burden; hence, it is crucial to identify a personalized plan for each patient with involvement of the multidisciplinary teams, including the cardiologist, cardiothoracic surgeon, and immunologist.

Although complete surgical excision was successful in case 1, this may not always be possible due to the degree of extention of tumor into critical structures nearby or the presence of a high tumor burden. An innovative approach was adopted in two neonates (Case 2 and 5) with multiple rhabdomyomas in the left ventricle causing critical LVOTO and severe LV failure. Simultaneous application of partial resection of left ventricular outflow tract tumor to provide immediate relieve of critical obstruction, a hybrid stage one procedure [17] including ductal stenting and bilateral pulmonary arteries banding to create a right ventricular assist systemic circulation, in the context of poorly performing left ventricle due to high tumor burden and increased afterload, and synergistic mTORi therapy were used to accelerate tumor regression.

**Fig. 5:**
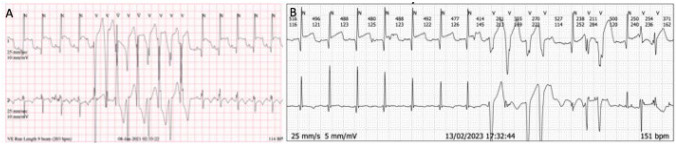
Serial Holter Strips in Case-5 **A** at initial presentation with frequent episode of non sustained ventricular tachycardia; **B** at 20-months after cessation of mTORi therapy showing frequent ventricular ectopic beats

Ductal stenting provided secure pulmonary blood flow in the immediate postnatal period [18], allowing time for mTORi to take effect in Case 3 proven to be a successful strategy and avoiding the need to subject the newborn to the risk of open heart surgery and cardiopulmonary bypass. A combination of mTORi and anti-arrhythmia was used in Case 4 with high-tumor burden producing mild-moderate multi-sites obstruction and frequent non-sustained ventricular arrhythmia (Fig. 5). Bespoke innovative approach on a case-by-case basis was proven effective in mitigating hemodynamic instability by alleviating cardiac obstruction and facilitating systemic cardiac output, allowing time for tumor to regress and restoring myocardial function.

Until recently, surgical resection was the only treatment option for symptomatic cardiac rhabdomyoma, along with other medical measures for stabilization and management of heart failure and arrhythmias. Surgical resection may be challenging to conduct in very small neonates with low birth weight and may not always be possible due to risk of injury to nearby structures. Furthermore, surgical resection may be associated with significant morbidity and mortality [7] compared to medical management.

Mammalian target of rapamycin inhibitors such as everolimus and sirolimus have been proven effective in the treatment of TSC manifestation in the brain, subependymal giant cell astrocytoma (SEGA), in kidneys angiolipoma (AML), and skin lesion [8, 10, 19]. Ever since the increased understanding of its role in curbing the overactivity of the mTOR pathway [7] and incidental discovery of potential therapeutic effect in cardiac rhabdomyoma in a child receiving mTORi for SEGA in 2011 [9], these agents have been increasingly accepted as effective therapeutic options for symptomatic cardiac rhabdomyoma. Several single-case reports and small series have demonstrated the efficacy and safety of mTORi therapy in inducing tumor regression in small neonates and infants with symptomatic cardiac rhabdomyoma [9, 11–15, 19–21]. We observed similar findings in our cohort. Four of the five cases in our series with TSC mutation showed good response to sirolimus, albeit at variable rates over different time intervals.

Interestingly, the infant who was negative for TSC mutation in our series failed to respond to sirolimus. The question whether treatment should be given to patients without the diagnosis of Tuberous sclerosis or before diagnosis is established remains unanswered. While identification of a pathogenic variant of the TSC1 or TSC2 is sufficient for the diagnosis or prediction of TSC regardless of clinical findings, between 10 and 15% of patients meeting diagnostic criteria for TSC have no mutation identified by conventional genetic testing [22, 23]. High-read depth approaches in next-generation sequencing (NGS) demonstrate low-level mosaic pathogenic variants in some of these individuals [22, 23]. To date, there are only a handful of case reports (4 everolimus, 1 sirolimus) showing positive response to mTORi in young children with negative TSC mutation [24–26]. While we understood the mechanism of action for mTORi in individuals with TSC mutation, there is a lack of understanding of pharmacodynamics (drug effect) of mTORi in TSC mutation-negative individuals with cardiac rhabdomyoma. This highlights the need for multicenter collaborative studies to evaluate the pharmacokinetics and pharmacodynamics of sirolimus, and their relation to genetics factors.

We chose to use sirolimus as first-line mTORi because unlike everolimus, it was available in liquid form for easy dispensing and administration in neonates and young infants. Although no studies have directly compared both drugs, there are some evidences to suggest that sirolimus induces tumor regression more rapidly than everolimus [9, 11, 13, 15]. Common mTORi side effects include stomatitis, dyslipidaemia, diarrhea, infections, and bone marrow suppression. In most cases, the side effects are mild and easily managed [8, 10, 27]. It is worth noting that side effects are dose dependent [28] and tend to decrease with time of the therapy [29]. Therefore, careful surveillance of these parameters is recommended. For example, at our institution, full blood count, renal, liver, and lipid profiles are performed at baseline prior to starting sirolimus and weekly with sirolimus serum level sampling until a therapeutic level is reached. All patients who are on sirolimus are prescribed prophylaxis antibiotic with co-trimoxazole to prevent opportunistic bacterial infections. Live vaccines are avoided, while the patients are actively being treated with sirolimus due to its immunosuppressive property. Generally, catch-up vaccinations will be performed for these patients at least 2 weeks after the completion of the sirolimus therapy [28]. Non-live vaccines can be administered according to local immunization schedule without changes in the mTORi therapy.

We observed a variable starting dose and serum level sampling time for mTORi at our center. The initial sirolimus dose varies from 0.9 mg/m^2^/day to 2.3 mg/m^2^/day, with a higher dosing prescription in the earlier cases compared with the latter (see Table 2). This may reflects an era effect, whereby the initial mTORi dosing was guided by the available literatures at the time and updated over time [11]. Serum drug levels were measured commonly on day seven of the treatment, and as early as day three. A target therapeutic range for mTORi between 15 and 20 ng/dL was chosen because it was believed to deliver therapeutic effects and avoiding toxicity. A recent systematic review by Sugalska et al. (2021) reported the efficacy and safety of mTORi in thirty case reports or case series involving 41 pediatric TSC patients with CRs: 68.3% (28/41) were treated with everolimus and 31.7% (13/41) with sirolimus. The authors observed that mTORi doses were significantly differed between these studies, especially in the sirolimus group [30]. This may be due to lack of high-quality pediatric data on mTORi in CR to date, and mTORi dosing in early case reports or series was extrapolated from studies conducted in adults or successful everolimus therapy in pediatric patients with SEGA [5, 8, 25]. Hence, large randomized studies such as the ORACLE trial may help to address this issue in the near future [31].

Neonates and young infants are predisposed to very high serum drug levels, because they have lower activity of liver enzymes. Their activity of CYP3A4 is approximately 30–40% compared to adults and children after first month of life, leading to lower drug clearances [32]. In our series, the four neonatal patients with supratherapeutic levels were commenced on sirolimus before seven days of life, while a 4-month-old infant with persistent low sirolimus level despite up-titration. In the 4 neonates, a starting sirolimus dose of 1 mg/m^2^/day, 1.4 mg/m^2^/day, and 2.3 mg/m^2^/day, respectively, generated a serum level of 47.8, 92, 26n, and 108 ng/mL. As for the 4-month-old infant, starting sirolimus dose of 0.9mg/m^2^/day yielded a level of 5.5 ng/mL. These findings confirmed there is a large variability in pharmacokinetics (PK) and pharmacodynamics (PD) in pediatric patients, for narrow therapeutic range drugs such as mTORi [33, 34]. One of four patients with supratherapeutic levels suffered acute renal failure, and elevated serum lipid level was observed in three of the four patients. Similar mild side effects were observed in the literature [11–13] and a case of pulmonary hemorrhage was noted in a neonate three days after everolimus therapy [35]. Mizuno et al. in 2017 reported on a PK model-based strategy for the precision dosing of sirolimus as part of prospective concentration controlled clinical trials in pediatric patients with vascular anomalies [36]. Twelve-month follow-up data were collected and analyzed from 52 pediatric patients, aged three weeks to two years old, and participating in a Phase 2 clinical trial [36]. The authors concluded that younger children such as neonates and young infants required lower sirolimus dose to achieve target level ~ 10ng/ml and they have lower sirolimus clearance compared to children older than two years of age. Based on their actual measured sirolimus concentrations over the twelve-month study period, to achieve level of ~ 10ng/mL for age groups 0–1, 1–2, 2–3, 3–4, 4–6, 6–9, 9–12, and 12–24 months, respectively, starting dose identified were 0.4, 0.5, 0.6, 0.7, 0.9, 1.1, 1.3, and 1.6mg/m^2^/day, respectively. Based on the evidence in the literature and the results from our series, we believe it is reasonable to consider a lower starting dose in small neonates and young infants and uptitrate according to real-time sirolimus level to the targeted therapeutic range (See the proposed strategy for sirolimus dosing and monitoring below).

The duration of treatment for our cohort was between seventeen days and four years. In general, the sirolimus was discontinued once 50–70% reduction in tumor size or alleviation of gradient was achieved, with exception in one case where the treatment was extended for 4 years with the intention to treat TSC-associated manifestation in the brain where the child suffered with intractable seizure. Similar approaches were observed in the reported cases in the literature [14, 37]. Rebound growth of the cardiac rhabdomyoma was observed in our cohort and in the literature after the cessation of sirolimus, and in some cases, mTORi was recommenced to mitigate the obstruction, however, in majority of the cases that was not necessary (11, 13).

There is a general lack of consensus in the literature concerning the prescribing strategy for mTORi, the target therapeutic range, and the timing for serum drug level sampling. We advocate for a standardized approach to mTORi prescription and monitoring to ensure the medication safety, especially in the small preterm or term neonates and infants who are more susceptible to drug toxicity due to their immature organ function and variable underlying pharmacokinetics.

We proposed the following strategy for sirolimus dosing and monitoring for neonates and infants:

1. Optimal starting sirolimus dose for neonates – 0.5mg/m^2^/day and titrate up by 10–15% based on serum drug level.
2. In premature neonates, to start at lower dose, e.g., 0.25mg/m^2^/day and uptitrate by 10–15% based on serum drug level.
3. Optimal starting sirolimus dose for infants 2 to 6 months old – 0.5 to 1mg/m^2^/day and uptitrate by 10–15% based on serum drug level.
4. Optimal starting sirolimus dose for infants 6 to 12 months old – 1 to 1.5mg/m^2^/day and uptitrate by 10–15% based on serum drug level.
5. Target therapeutic serum sirolimus level – 15 to 20 ng/mL
6. Timing of serum level monitoring (Trough) – day 3 and day 7 post-1st dose and then weekly once the steady state is achieved. Repeat this step after any dose adjustment.
7. Duration of treatment – aim for 50–70% reduction in tumur size or alleviation of gradient, e.g., 4 to 8 weeks or longer if indicated for synergistic therapy of other TSC manifestations (e.g., refractory seizures)
8. Close monitoring for rebound growth of CR upon cessation of mTORi therapy, e.g., twice weekly TTE initially

## Conclusion

Treatment of symptomatic rhabdomyoma requires a bespoke treatment strategy for each patient based on the underlying pathophysiology, with MDT involvement of cardiologists, cardiothoracic surgeons, intervention cardiologists, oncologists, and immunologists. Although mTORis have been proven effective in accelerating regression of rhabdomyomas, the prescription and monitoring guide will ensure a more standardized approach and safety in the symptomatic CR patients. As resistance to mTORi treatment remains poorly understood, whether due to their genetic makeup or underlying pharmacokinetics and pharmacodynamics, further studies are warranted, ideally involving international multicenter collaborations.

## Data Availability

No datasets were generated or analysed during the current study.
